# Bowel Gangrene Is a Rare but Dreaded Complication of Aluminum Phosphide Poisoning

**DOI:** 10.7759/cureus.27885

**Published:** 2022-08-11

**Authors:** Akshita Chandel, Dheeraj Dheeraj, Jithesh G, Mukesh Bairwa

**Affiliations:** 1 Internal Medicine, All India Institute of Medical Sciences, Dehradun, IND

**Keywords:** gastrointestinal hemorrhage, poisoning, bowel gangrene, phosphine gas, celphos, aluminum phosphide

## Abstract

Poisoning is a significant contributor to mortality and morbidity throughout the world, and one of the most common pesticide poisonings is organophosphates, followed by phosphides. Ingestion of aluminum phosphide can induce severe gastrointestinal irritation leading to hemorrhage and ulcerations. Gastrointestinal ischemia, gangrene, and hemorrhage in the ileum secondary to aluminum phosphide poisoning have not been reported in the literature. The authors report a case of an 18-year-old man who had consumed 10 grams of Celphos, aluminum phosphide powder. The patient developed lower gastrointestinal ischemia and hemorrhage due to the direct effect of aluminum phosphide, leading to bowel gangrene. The gangrenous segment caused fecal peritonitis and sepsis, leading to multiorgan failure and death. This case report emphasizes the significance of the corrosive nature of aluminum phosphide; lower gastrointestinal hemorrhage is a rare but fatal complication of aluminum phosphide poisoning.

## Introduction

Pesticide poisoning is a global problem that accounts for significant morbidity and mortality. Each year around 3,00,000 deaths occur from self-inflicted pesticide poisoning [1]. Pesticide self-poisoning accounts for around a third of all suicides worldwide, making it the most frequently used method of suicide [2].

Aluminum phosphide (ALP) is one of the most extensively used pesticides to protect stored products and crops but is highly toxic to humans. Data have revealed an increasing incidence of poisoning due to ALP [3]. ALP is the second most common agricultural pesticide, the first being organophosphates, in India [3].

ALP generates highly toxic phosphine gas when exposed to the moist surface of the respiratory tract and gastrointestinal (GI) tract. Phosphine inhibits vital enzymes and the electron transport chain, decreasing adenosine triphosphate production and increasing reactive oxygen species production. The earliest and most typical manifestations are GI tract symptoms like retrosternal burning, epigastric pain, and vomiting. Cardiovascular includes circulatory failure, and severe hypotension are prevalent features and the most important cause of mortality [4-6].

The local corrosive action of ALP may lead to extensive hemorrhages and ulceration along the GI tract [7]. Upper GI hemorrhages and esophageal stricture formation have been observed in cases of ALP poisoning [8]. However, lower GI involvement in the form of hemorrhage and bowel ischemia has not yet been reported in the literature.

Acute poisoning with ALP is a global problem, primarily encountered in the Indian subcontinent. The mortality is remarkably high because of multiple factors like a lack of complete understanding of kinetics and a successful antidote. Death usually results within 24 hours of ingestion due to cardiotoxicity. Delayed death can be due to multiple organ failures. The corrosive action of ALP and phosphine gas generation may lead to extensive hemorrhage and ulcerations in the GI tract.

The objective of this case report is to explain the severe lower GI involvement in a case of ALP ingestion, which manifested as a major GI hemorrhage, small bowel ischemia, and gangrene.

## Case presentation

An 18-year-old man without any comorbidity presented with complaints of nausea and multiple episodes of vomiting. The family provided a history of intentional ingestion of one pouch of Celphos containing 10 grams of 56% ALP. Following ingestion, he had multiple episodes of vomiting. He was taken to the hospital after six hours of ingestion, where he was treated conservatively with intravenous fluids and pantoprazole infusion and was referred to a tertiary care center. He was admitted to the intensive care unit with a heart rate of 102/min, respiratory rate of 22/min, blood pressure of 104/70 mmHg, and temperature of 98.4 F. On hospital day (HD) 2, he started complaining of dark, red-colored bleeding per rectum along with gradual abdominal distension and pain in the abdomen.

**Figure 1:**
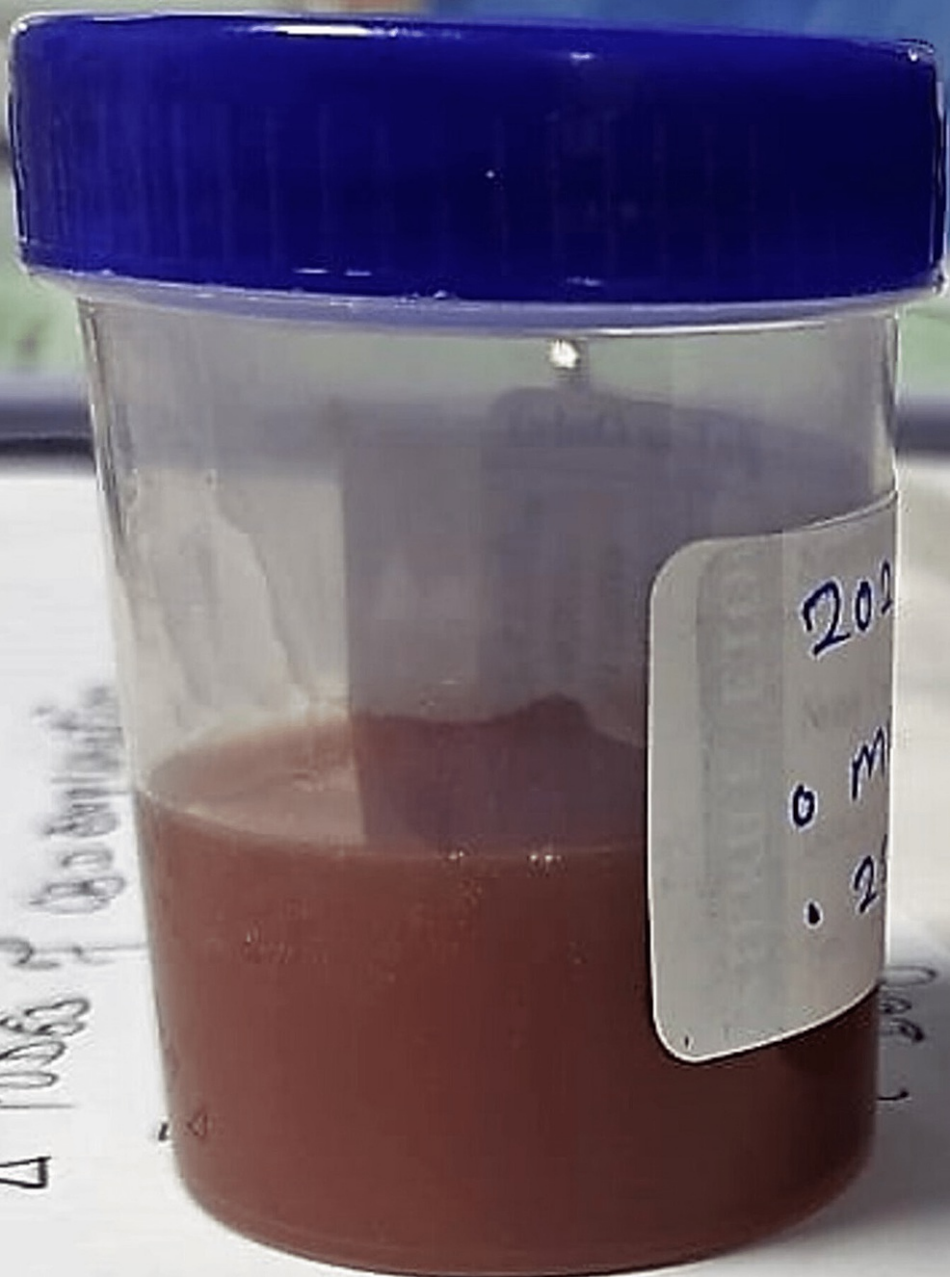
Diagnostic ascitic tap showing muddy brown fluid

Local rectal causes of bleeding were ruled out by digital rectal examination and proctoscopy. His hematochezia continued, for which upper GI and lower GI endoscopy were performed on HD 3, which revealed linear esophageal ulcers, diffuse mucosal erythema, and mucosa stained with blood, respectively. However, upper and lower GI endoscopy didn't identify the bleeding source. His abdominal distension was progressive, and on HD 4, an abdominal ultrasound was performed, which was suggestive of gross septate ascites with features suspicious of perforation peritonitis. An erect abdominal X-ray didn't demonstrate pneumoperitoneum, which showed distended bowel loops and no air under the diaphragm. Abdominal paracentesis was performed, which was grossly muddy brown in color, as shown in Figure 1. Biochemical analysis of ascitic fluid revealed 2500 cells/µL and satisfied Runyon's criteria for secondary bacterial peritonitis [9]. The patient was started on intravenous antibiotics cefotaxime and metronidazole.

Hematochezia persisted, and the patient had a significant drop in hemoglobin, which was 15.1 g/dl on HD 1 and 8.6 g/dl on HD 4. The patient's blood investigations also revealed a rising trend in total leucocyte counts (12000 on HD1 and 19080 on HD2) and worsening liver (total bilirubin level was 1.7 mg/dl on HD 1 and 3.7 mg/dl on HD 4) and kidney functions (serum creatinine was 1.2 mg/dl on HD 1 and 4.9 on HD 4). The patient showed an increasing trend of lactate levels in blood gas analysis, which was 2.0 mmol/L on HD 1 and 16.2 mmol/L on HD 2, favoring bowel ischemia [10]. Contrast-enhanced computed tomography abdomen with oral positive contrast was done on HD 5, which was unremarkable.

**Figure 2:**
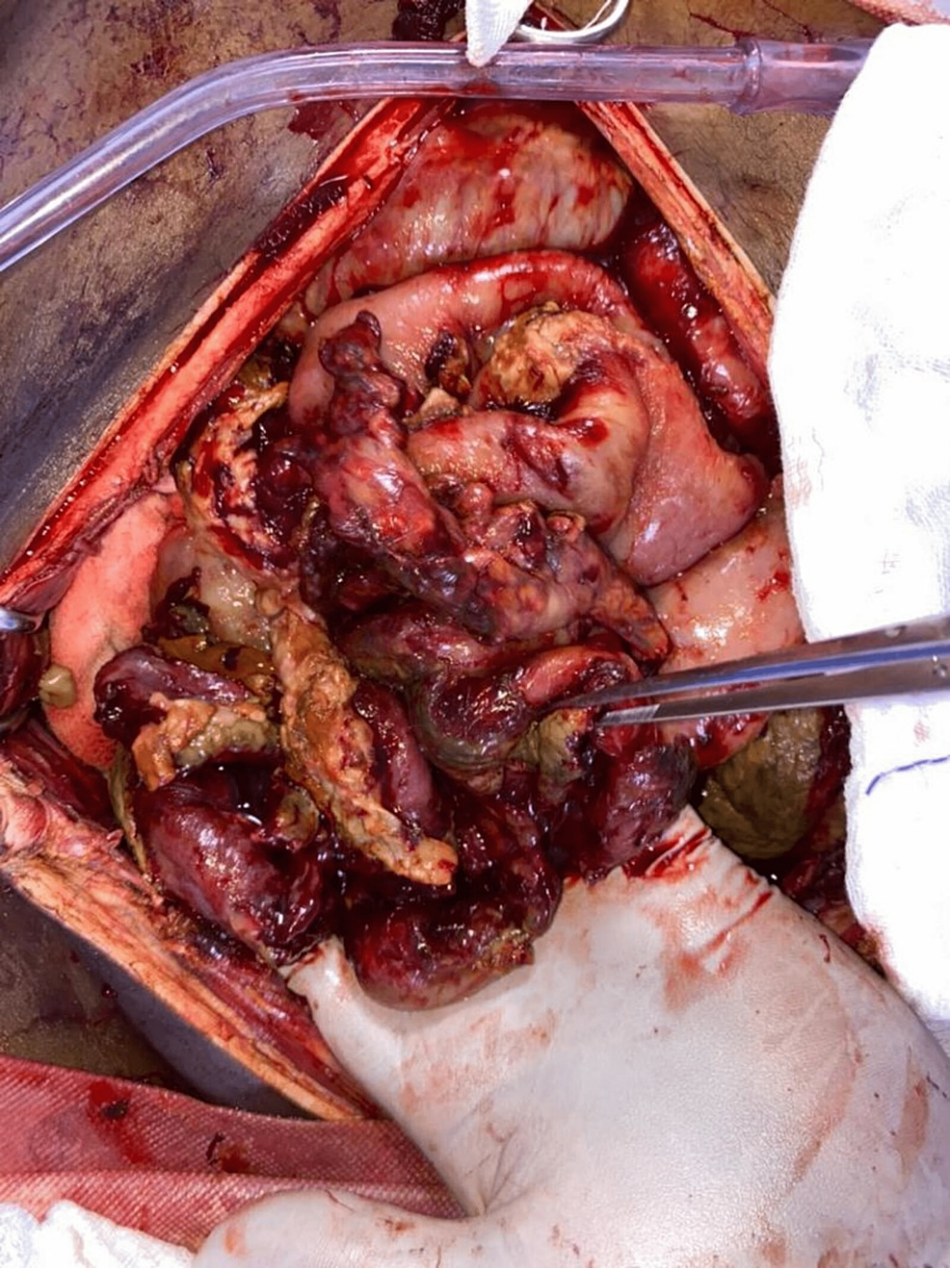
Exploratory laparotomy revealing a gangrenous sloughed off ileal segment

General Surgery consultation was sought because of secondary bacterial peritonitis, and the patient was taken for midline exploratory laparotomy on HD 6. Surgical findings were a gangrenous sloughed off ileal segment and fecal peritonitis, as shown in Figure 2. Bowel resection was performed with a double-barrel loop ileostomy. However, the patient deteriorated over time and developed shock, for which he was administered ionotropic support, norepinephrine as the first-line agent, and antibiogram-based antibiotics intravenous meropenem. The patient's ionotropic requirement increased gradually, and on day 11, the patient developed cardiac arrest and could not be resuscitated.

## Discussion

The severity of ALP poisoning depends on the dose, formulation, route of administration, old exposed formulations or new packed formulation, and the time between ingestion and initiation of resuscitative efforts. ALP poisoning mortality rates vary from 40% to 80% [11]. ALP, upon exposure to water, releases phosphine gas, which inhibits mitochondrial cytochrome oxidase and oxidative respiration. This leads to the generation of reactive oxygen species, which causes toxicity due to oxidative stress and direct corrosive damage [12].

Thus, the absorption of phosphine gas causes damage to internal organs. Also, aluminum phosphide does have caustic effects on the GI system, which is often overlooked. Kochhar et al. studied the clinical profile of ALP-induced esophageal strictures and suggested that strictures result from local injury and are usually caused by exposed tablets [13]. Nijhawan et al. further elaborated that ALP ingestion leads to esophageal stricture and is resistant to endoscopic dilation [14].

Although ALP-related strictures in the esophagus are reported, corrosive effects leading to fatal complications are not found in the literature [15]. This case report emphasizes a unique case of ALP ingestion leading to fatal lower GI hemorrhage.

The patient developed bowel gangrene and lower GI hemorrhage, leading to refractory shock. Blood investigations suggested a persistent fall in hemoglobin, an extreme rise in lactate levels, worsening liver and kidney functions, and a rise in leukocyte counts. Ascitic fluid analysis was suggestive of secondary bacterial peritonitis. The fall in hemoglobin was due to persistent GI hemorrhage from ulceration and necrosis of the ileum, as evidenced during laparotomy. Secondary bacterial peritonitis was due to ileal gangrene, which might have occurred due to the direct toxic effect of ALP and bowel ischemia.

Death in ALP poisoning usually occurs within the first 24 h due to acute cardiotoxicity. Other causes of death in a person surviving for more than a day include adult respiratory distress syndrome, upper GI hemorrhage, metabolic disorders, and liver failure. Both metal phosphides and phosphine have corrosive actions, which play a role in causing extensive hemorrhage and ulcerations in the GI tract [7]. However, lower GI hemorrhage associated with ALP poisoning has not been reported, which, in this case, led to the mortality of our patient. Gangrenous ileal segment, hemorrhages, and fecal peritonitis were noted during surgery. A direct mucosal injury could have led to gangrene of the ileal segment with hemorrhage from that site, leading to a persistent fall in hemoglobin. The gangrenous segment further led to fecal peritonitis.

This case provides insight to consider ALP as a cause of bowel ischemia and gangrene and thus necessitates the need to consider early operative intervention in patients with ALP poisoning presenting as GI hemorrhage and extreme lactate elevation. Also, this case report encourages the use of blood lactate levels as an index of the severity of ALP poisoning [16].

## Conclusions

Although ALP-related strictures in the esophagus are reported, corrosive effects on the lower GI leading to fatal complications are not reported in the available literature. The authors report this case given the rarity of available literature that suggests the corrosive nature of ALP on the lower GI tract in a victim who survives the acute period of poisoning. Lower GI bleeding is a rare but fatal complication of ALP poisoning. An extreme rise in serum lactate levels should lower the threshold for diagnosing bowel ischemia and undergoing early surgical intervention in ALP poisoning.
